# Successive Norovirus Outbreaks at an Event Center — Nebraska, October–November, 2017

**DOI:** 10.15585/mmwr.mm6828a2

**Published:** 2019-07-19

**Authors:** Rebecca J. Free, Bryan F. Buss, Samir Koirala, Monica Ulses, Anna Carlson, Brianna Loeck, Tom Safranek

**Affiliations:** ^1^Epidemic Intelligence Service, CDC; ^2^Division of Public Health, Nebraska Department of Health and Human Services, Lincoln, Nebraska; ^3^Career Epidemiology Field Officer Program, Division of State and Local Readiness, Center for Preparedness and Response, CDC; ^4^Sarpy/Cass Health Department, Papillion, Nebraska.

In October 2017, the Nebraska Department of Health and Human Services (NDHHS) was notified by a local health department of a gastrointestinal illness outbreak among attendees of a wedding reception at facility A, an event center. Shortly thereafter, state and local public health officials began receiving reports of similar gastrointestinal illness among attendees of subsequent facility A events. An investigation was initiated to identify cases, establish the cause, assess possible transmission routes, and provide control recommendations. Overall, 159 cases consistent with norovirus infection (three confirmed and 156 probable) were identified among employees of facility A and attendees of nine facility A events during October 27–November 18, 2017. The investigation revealed a public vomiting episode at the facility on October 27 and at least one employee involved with preparing and serving food who returned to work <24 hours after symptom resolution, suggesting that a combination of contaminated environmental surfaces and infected food handlers likely sustained the outbreak. Recommendations regarding sanitation and excluding ill employees were communicated to facility A management. However, facility A performed minimal environmental cleaning and did not exclude ill employees. Consequently, transmission continued. To prevent persistent norovirus outbreaks in similar settings, public health officials should ensure that involved facilities implement a comprehensive prevention strategy as early as possible that includes extensive sanitation and strict exclusion of ill food handlers for at least 48 hours after symptom resolution (*1*).

## Investigation and Results

On October 30, 2017, public health officials became aware of approximately 30 persons who developed gastrointestinal illness after attending a wedding reception (event 1) on October 27 at facility A. Norovirus was suspected based on ill attendees’ reports of developing diarrhea, vomiting, abdominal cramps, and fever approximately 12–48 hours after the event. On November 6, investigators learned of similar gastrointestinal illness among attendees at five subsequent facility A events (events 2–6), at which point an Internet-based questionnaire that assessed symptom history, events attended, and food items consumed was developed. E-mail addresses for facility A employees were provided by facility management. Investigators worked with event organizers to disseminate the questionnaire to attendees of the first six events held at facility A during the investigation period, as well as four subsequent events that were also ultimately affected by the outbreak. A case-control study was performed. A probable case was defined as the occurrence of diarrhea (≥3 loose stools within 24 hours) or vomiting and at least one other symptom (nausea, abdominal cramps, diarrhea, or vomiting) in a facility A employee or an event attendee who reported illness onset 6–72 hours after attending a facility A event on or after October 27. Confirmed cases met the probable case definition and had norovirus RNA detected in a stool specimen by real-time reverse transcription–polymerase chain reaction (RT-PCR) (*2*). Controls were identified as facility A employees who were not ill and were exposed to facility A during the study period or event attendees who were not ill and attended an event during the study period. Estimated attack rates (ARs) were calculated per event, using host-estimated number of attendees as denominators.

Ten events that included food service provided by facility A were held at the facility during October 27–November 18, 2017. Overall, 378 persons from nine events completed questionnaires, including 18 of 25 (72%) employees and 360 of 1,383 (26%) event attendees (Table). Only one questionnaire response among 70 attendees was received for the tenth event and was thus excluded from analysis. Overall, 159 persons (six employees and 153 event attendees) reported illness meeting the probable (156) or confirmed (three) case definition (Figure); 186 controls were identified. Comparison of food items consumed by case-patients and controls was limited because the only items available at all nine events were water, ice, and drink garnishes; however, no item was significantly associated with illness. Estimated ARs for the first six events, which occurred before any public health intervention, ranged from 7% to 35% per event (median = 18.5%) (Table).

**TABLE:** Attendee questionnaire response rates and estimated gastroenteritis attack rates per facility A event — Nebraska, October–November 2017

Facility A event	Event date	Estimated no. of attendees*	Total no. (%) of respondents	Cases	Estimated attack rate (%)
Event 1	Oct 27	115	43 (37)	33^†^	29
Event 2	Oct 28	130	42 (32)	22	17
Event 3	Oct 28	20	13 (65)	7	35
Event 4	Nov 2	10	2 (20)	2	20
Event 5	Nov 3	120	24 (20)	18	15
Event 6	Nov 4	128	16 (13)	9	7
Event 7	Nov 10	150	46 (31)	6	4
Event 8	Nov 11	360	127 (35)	53^§^	15
Event 9	Nov 18	350	47 (13)	3	1
**Total**	**—**	**1,383**	**360** (**26**)	**153**	**11**

* Estimated from lists provided by event hosts.

^†^ Includes two laboratory-confirmed cases.

^§^ Includes one laboratory-confirmed case.

**FIGURE:**
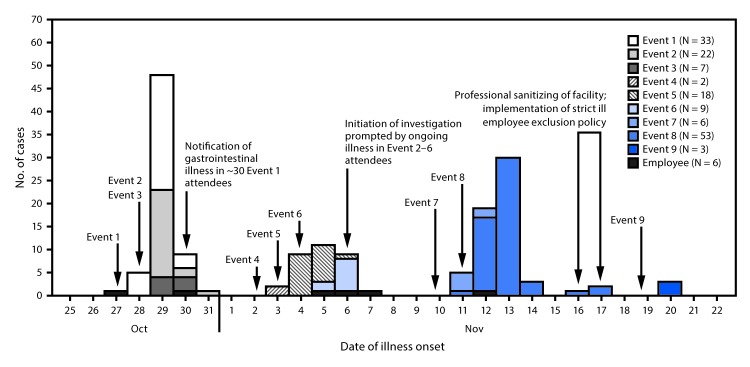
Probable and confirmed cases of norovirus gastroenteritis associated with facility A event attendees (N = 153) and employees (N = 6), by event and illness onset date*,^†^ — Nebraska, October–November 2017 * One laboratory-confirmed norovirus case on October 29, 2017, October 30, 2017, and November 13, 2017. ^†^ One employee returned to work <24 hours after symptom resolution on November 7, 2017.

The investigation uncovered a witnessed episode of vomiting in a public area near the event space by an event attendee. The episode occurred at the beginning of the October 27 event (event 1) on carpeting in the lobby at the entrance to the event hall and might have represented the initial introduction of norovirus into facility A. Although no testing of environmental surfaces was conducted to confirm, it is possible this vomiting contaminated environmental surfaces.

On November 7, investigators learned that the carpeting where vomiting occurred on October 27 had been swept with a vacuum cleaner and inadequately sanitized; the agent used did not have efficacy against norovirus. Investigators recommended sanitizing environmental surfaces with a sodium hypochlorite (chlorine bleach) solution or a disinfectant specifically registered by the Environmental Protection Agency (EPA) as effective against norovirus*^,†^ and excluding ill employees from work until ≥48 hours after symptom resolution (*1*). However, cases of gastroenteritis occurred at two events that were held on November 10 (event 7) and 11 (event 8) after these recommendations were made; estimated ARs at event 7 and event 8 were 4% (six of 150 attendees) and 15% (53 of 360 attendees), respectively, indicating ongoing transmission. Investigators subsequently learned of an employee who left work when she became ill at 10:00 a.m. on November 7, with nausea, vomiting, fever, headache, and myalgias, and returned to work preparing and serving food on November 8, <24 hours later.

Stool specimens from three ill persons were tested. Norovirus genogroup II was detected by real-time RT-PCR from all three stool specimens tested; further genetic sequencing by Nebraska Public Health Laboratory and CDC confirmed that all three specimens yielded the same norovirus genotype, GII.P12-GII.3. Two of the case-patients in whom norovirus was laboratory-confirmed attended the October 27 event (event 1), and the third attended the event on November 11 (event 8).

## Public Health Response

After initial public health recommendations to use disinfectants registered by the EPA and exclude ill employees failed to halt transmission (*1*), several discussions were held with facility A management during the period leading up to a planned event on November 18 (event 9). The recommendation for strict employee exclusion was reiterated on November 15, along with ideas for minimizing pressures on employees to work while ill, such as offering paid sick leave and bringing in staff members from a different location to work the event. Consideration was given to postponing the upcoming event or finding an alternative location for it. Facility A hired a professional cleaning service experienced with norovirus eradication to sanitize the facility on November 16 and 17. After thorough sanitation and strict employee exclusion were implemented, the event held on November 18 (event 9) had an estimated AR of 1% (three of 350 attendees), indicating reduced transmission (Table). No further illnesses in facility A employees or event attendees were reported to public health officials.

## Discussion

Norovirus, the most common cause of outbreak-associated acute gastroenteritis worldwide, is highly efficient at causing human disease (*3*). The virus is extremely contagious, with a low infectious dose capable of causing infection with as few as 18–2,800 virus particles (*4*,*5*). In addition, large numbers of virus can be shed by infected persons, even those with asymptomatic infections (*1*). Norovirus is resistant to many common commercial disinfectants and is able to persist on environmental surfaces for up to 2 weeks (*6*).

Transmission occurs through several different routes, and multiple transmission routes can coexist during norovirus outbreaks (*6*,*7*). In addition to foodborne and direct person-to-person spread, transmission can also occur through ingestion of aerosolized particles and through contact with contaminated environmental surfaces, which are believed to harbor the virus and play a role in sustaining outbreaks (*8*,*9*). Multiple outbreaks caused by foodborne sources and subsequently perpetuated by environmental contamination or person-to-person spread have been described (*7*,*10*). In addition, when contaminated food items are implicated in outbreaks, infected food handlers are often involved (*1*).

In this setting of successive outbreaks at the same event center, norovirus was likely transmitted through a combination of persistently contaminated environmental surfaces and ill food handlers (*7*). The investigation findings indicate that the initial public vomiting episode likely contaminated the carpeting at the entrance to the event hall. Inadequate sanitizing of the area and aerosolization of the virus resulting from subsequent vacuuming could both have led to further spread. Although no environmental testing was done, investigators suspect that widespread environmental contamination was likely present (*9*). Transmission was halted only after the facility was thoroughly cleaned and a strict ill employee exclusion policy was enforced.

The findings in this report are subject to at least two limitations. First, because the total number of attendees at each facility A event was not known, investigators had to rely on host estimations. Accordingly, calculation of exact ARs was precluded. Similarly, questionnaire distribution to individual attendees was facilitated by each event’s host. As a result, investigators had no way of knowing how many attendees successfully received the invitation to complete the Internet-based questionnaire, and accuracy of corresponding AR calculations might have been affected. Because methodology for calculating ARs was consistent across all events, the potential of adversely affecting comparison of event-specific ARs was likely limited. However, the limitation was believed to introduce enough bias to preclude a cohort analysis. Second, environmental sampling that might have helped elucidate possible transmission routes was not done. By the time public health officials learned of the outbreak’s ongoing nature, 10 days had passed since the initial public vomiting episode. Because results of environmental testing would not have changed the recommendation for extensive sanitation, such testing was not prioritized.

Mitigation efforts for ongoing norovirus outbreaks in similar settings should include a comprehensive prevention strategy that attempts to address all possible routes of norovirus transmission. In this setting, control measures that included extensive environmental decontamination and strict exclusion of all ill food handlers for ≥48 hours after symptom resolution were needed to halt the outbreak. Public health officials can also verify that facilities involved in similar persistent outbreaks are implementing recommended public health interventions.

**Summary**

**What is already known about this topic?**

Norovirus, an extremely contagious cause of gastroenteritis, can be transmitted by infected food workers and is difficult to remove from contaminated surfaces.

**What is added by this report?**

An investigation into an ongoing gastrointestinal illness outbreak identified 159 persons reporting illness meeting the case definition; laboratory testing confirmed norovirus cases. Public health recommendations were not strictly followed, and transmission continued for approximately 2 weeks. Halting transmission required a coordinated approach involving thorough environmental decontamination and a strict ill employee exclusion policy.

**What are the implications for public health practice?**

Mitigation efforts for ongoing norovirus outbreaks in similar settings should include a comprehensive prevention strategy that addresses all possible routes of norovirus transmission.

## Footnotes

* https://www.epa.gov/sites/production/files/2018-04/documents/list_g_disinfectant_list_3_15_18.pdf.

† Per CDC guidance, “this list should be interpreted with caution because the efficacy of these products is determined by using the surrogate feline calicivirus, which exhibits different physiochemical properties than human norovirus and therefore might not reflect a similar disinfection efficacy profile.” https://www.cdc.gov/mmwr/preview/mmwrhtml/rr6003a1.htm.
